# Effect of a high bicarbonate mineral water on fasting and postprandial lipemia in moderately hypercholesterolemic subjects: a pilot study

**DOI:** 10.1186/1476-511X-12-105

**Published:** 2013-07-18

**Authors:** Yassine Zair, Fatima Kasbi-Chadli, Beatrice Housez, Mathieu Pichelin, Murielle Cazaubiel, François Raoux, Khadija Ouguerram

**Affiliations:** 1 L'Institut du thorax, IRT, 8 quai Moncousu, BP 70721, Nantes Cedex 1 44007, France; 2 BIOFORTIS, 3 route de la Chatterie, Saint-Herblain 44800, France; 3 Multimed, villa Lantiez, Paris 75017, France; 4 CRNH, Human Nutrition Research Center of Nantes, CHU, Nantes F-44093, France

## Abstract

**Background:**

During postprandial state, TG concentration is increasing and HDL cholesterol decreasing, leading to a transitory pro-atherosclerotic profile. Previous studies have reported that bicarbonate water improve postprandial lipemia. The objective of this study was to analyze the effect of a strongly bicarbonated mineral water on lipoprotein levels during fasting and postprandial state.

**Methods:**

A controlled, randomised, double-blind cross-over design was conducted in 12 moderately hypercholesterolemic subjects after a daily ingestion of 1.25 L of mineral (SY) or low mineral water during eight weeks separated by a one week wash-out period. Blood samples were collected in first visit to the hospital (V1) before water consumption (referent or SY) and in a second visit (V2) after eight week water consumption period. The effect of the consumed water was studied in fasting and in postprandial state during ingestion of a meal and 0.5 L of water.

**Results:**

Comparison of data between V1 and V2 after SY consumption showed a significant decrease in triglyceridemia (23%), VLDL TG (31%) and tendency to a decrease of VLDL cholesterol (p = 0.066) at fasting state. Whatever the consumed water during postprandial state, the measurement of total areas under curves did not show a significant difference. No difference was observed between SY and referent water consumption for measured parameters at fasting and postprandial state.

**Conclusion:**

When subjects consumed SY we showed a decrease of their basal TG and VLDLTG. The unexpected absence of effect of high mineralized water on postprandial lipemia, probably related to experimental conditions, is discussed in the discussion section.

## Background

Prolonged postprandial hyperlipidemia constitutes an important cardiovascular risk factor [1,2]. This risk is related to both increased triglyceride (TG)-rich lipoprotein (TRL, chylomicrons, remnant of chylomicrons and VLDL) levels and decreased HDL cholesterol. The decrease of HDL cholesterol often associated with an increased TRL [3] and is considered as atherogenic [4] through the implication of these lipoproteins in the transport of excess cholesterol from peripheral tissues to the liver, a process known as reverse cholesterol transport [5].

Improving postprandial lipid profile on the basis of reducing TRL and increasing HDL cholesterol is considered as a good strategy to fight the development of cardiovascular disease. Therefore, dietary recommendations advise the reduction of saturated fat consumption to decrease atherogenic lipoproteins in fasting plasma and reduce the risks of cardiovascular disease [6,7].

Beverages are essential components of the diet and may also influence the levels plasma lipids. Of note, investigations of mineral waters in animal models presented conflicting [8,9]. A first study reported that, in fasting state, the consumption of Calcium/magnesium rich waters decreased plasma total cholesterol and LDL cholesterol in rat [9] while another study showed an increase in HDL cholesterol and stabilization of LDL cholesterol in rat fed cholesterol rich diet, with an active conversion of cholesterol into bile acid [10]. Investigations in human also reported some positive effects of mineral water consumption. Study in human showed also that mineral water decreased total and LDL cholesterol in plasma [11]. Sulphur water oral administration (Wiesław water, Poland) in patients with atheroslcerosis showed a decrease in total and LDL cholesterol and TG [12]. Also, in pioneer experiments Capurso *et al*. [13] have shown that the mineral bicarbonated water (Montecatini, Italy) presented decreasing effect on total and LDL cholesterol in fasting state. Similar effects have been reported for bicarbonated water from another source (Vichy Catalan, Spain) [14-16]. This justifies recommendation of such waters in hypercholesterolemia. However, the compositions of minerals in the carbonated waters used in these studies were not comparable and in some case not fully known [17], preventing generalization for the use of bicarbonated mineral waters. Moreover, most of these studies were interested by the changes induced by mineral waters on LDL cholesterol, while the effects on TG and HDL of hypercholesterolemic subjects were inconsistently shown [14,15]. Of note, Schoppen et al. [16] reported a reduced postprandial lipemia in healthy postmenoposal women with mineral water rich in bicarbonate probably related to an increase of intestinal pH which is known to affect lipid absorption [18]. Improvement of postprandial lipemia was also reported in healthy normocholesterolemic young subjects, and the effect was linked to a decrease in cholescystokinin (CCK) level [19,20].

Moreover, mineral carbonated waters present very variable composition in total mineralization and precise ionic profile [17], preventing generalization for the use of bicarbonated mineral waters.

Based on this background we investigated the effect of strongly mineralized bicarbonate water (Saint-Yore, France) in both fasting and postprandial state on TG and cholesterol lipoprotein profile.

## Subjects and methods

The experimental protocol was approved by the Ethical Committee of Nantes (CPP Ouest IV) and performed in accordance with Helsinki Declaration of 1975, as revised in 1983. Each subject received a complete explanation of the investigation and gave an informed written consent before the beginning of the study. No subject withdrew during the study.

### Subjects

Twelve men aged 20–60 years, with BMI 18.5 to 25 kg/m^2^ and diagnosed with moderate hypercholesterolemia (from 2.20 to 3 g/L) participated in the study. A medical history, a physical examination and a routine laboratory blood screening (glucose, cholesterol, TG, transaminases, creatinine levels, thyroid function and renal clearance) were performed before inclusion. Subjects suffering from type 2 diabetes, transit or metabolic disorders or high blood pressure or treated with hypolipidemic drugs were excluded. The participants were instructed not to deviate from their regular habits during the study.

To assess average food intakes during the study, a food-frequency questionnaire was administered to the participants. Participants are also given a questionnaire and a list of all aliments and asked to note precisely each aliment and the amount consumed for 3 days. Analysis of the questionnaire data by dietitian provided estimates of consumption frequency and the nutrients composition of meals for 3 days per week (two days from Monday to Friday and one day in the week-end) during the eight weeks of each period. Thus, the background diets provided 15% of energy from proteins, 50% of energy from carbohydrates, and 35% of energy from fat.

### Experimental protocol

The study was conducted according to a controlled, randomised, double-blind cross-over design with two 8-week experimental periods separated by a 1-week wash-out period.

During the experimental periods, subjects had to consume daily 1.25 L of one of the 2 tested sparkling waters: Saint-Yorre (SY) or Ogeu (referent). SY water is characterized by a high mineral content unlike referent water (Table 1). Subjects were randomly assigned to one of the two sequences of product consumption (SY/referent or referent/SY) following their inclusion in the study.

**Table 1:** Mineral content of the studied waters: units mg/L (mmoles/L in brackets)

**Mineral content**	**SY**	**Referent**
	**water**	**water**
Bicarbonates	4168 (68.3)	183 (3.0)
Chlorides	329 (9.3)	48 (1.4)
Sulfates	186 (1.9)	18 (0.2)
Calcium	85 (2.1)	48 (1.2)
Magnesium	11 (0.4)	12 (0.5)
Sodium	1626 (70.7)	31 (1.3)
Potassium	117 (3.0)	1 (0.02)
Nitrates	2 (0.03)	5 (0.08)

Five visits were planned at the clinical center for each subject: the pre-inclusion visit (V0), at the beginning of the experimental periods (V1) and on the last day of the experimental periods (V2). The baseline characteristics of study participant are shown in Table 2.

**Table 2:** Baseline characteristics of study participants

	**Mean (SD)**
Age (years)	40.6 (± 9.4)
Height (cm)	175.1 (± 5.8)
Body mass index (kg/m^2^)	23.1 (± 2.2)
Glucose (g/L)	0.948 (± 0.074)
Plasma cholesterol (g/L)	2.434 (± 0.213)
LDL cholesterol (g/L)	1.29 (±0.20)
Plasma triglycerides (g/L)	0.970 (±0.317)
Systolic blood pressure (mmHg)	119.8 (± 13.3)
Diastolic blood pressure (mmHg)	79.7 (± 13.6)

Mean±SD, n=12.

Experimental sessions were performed on V1 and V2 of both periods. On each occasion, fasted subjects came to the clinical center in the morning. After medical examination, an intravenous catheter was inserted into an arm vein and a first blood sample was collected (T0). Then, the subject consumed a standardised meal (Table 3). This meal provided 798 Kcal and constituted with 37.7 g of fat, 89.20 g of carbohydrate and 25.72 g of protein with 0.5 L of the sparkling water prescribed during the experimental period in progress. Additional blood samples were obtained at T30min, T1h, T2h, T3h, T4h, T6h and T8h to determine the total area under curve (TAUC). Subjects were instructed not to eat or drink anything else during the 8-h experimental session.

**Table 3:** The composition of standard meal given during postprandial test

	**Amount (g)**	**Proteins (g)**	**Carbohydrates (g)**	**Lipids (g)**	**Energy (Kcal)**
Butter	20	0.14	0.08	16.6	150.28
Cheese	60	17.64	0.12	17.28	226.56
stewed apricot	220	1.54	50.6	0.22	210.54
white bread	80	6.4	38.4	3.6	211.6
energy (Kcal)		102.88	356.8	339.3	798.98
% of total energy		12.88	44.66	42.47	100

### Data collection

Data were analyzed at T0 (chronic effect of water consumption) and as area under the curve (acute effect of water consumption) for plasma (cholesterol, TG, glucose and insulin), apolipoprotein A1 in HDL and free cholesterol, cholesteryl ester, total cholesterol and TG in all the lipoproteins.

Before starting blood pressure assessment, the healthy subjects had to rest in a horizontal position for 15 minutes. A blood pressure cuff was applied on the contra lateral upper arm.

### Biochemical analysis

Blood was collected into tubes containing EDTA for determination of all lipid parameters. For glucose measurement blood was collected into tubes containing fluoride which inhibit glycolytic pathway [21].

Total cholesterol, unesterified cholesterol, cholesteryl ester, TG and glycemia were assayed by enzymatic colorimetric test with Hitachi 911; insulinemia by ELISA (DakoCytomation Insulin kit); and A1 by immunoturbidimetric assay with Hitachi 911.

#### Lipoprotein separation

Isolation of lipoproteins was undertaken by using ultracentrifugation (CO-LE80K, rotor 50.4 TI; Beckman Coulter Inc., Brea, CA, USA). After overlaying 2 mL of serum with 4 mL of NaCl solution with a density of 1.006 and centrifuged for 23 minutes at 19040 g and 10°C, the chylomicrons were first isolated from the meniscus of the tube. The lower layer was overlaid with 2 mL of NaCl solution with a density of 1.006 and centrifuged for 18 hours at 246758 g and 10°C to obtain VLDL fraction. After removal of the supernatant, LDL (1.020 < d <1.063) and HDL (1.020 < d <1.063 g/mL) were respectively separated by standard sequential ultracentrifugation methods [22].

### Statistical analysis

A statistical power of 80% was calculated for 10 participants. The results are expressed as means ± SDs from all 12 participants. The postprandial response of all measured parameters was calculated as total area under the curve, ie, the total increase above zero data for 480 min.

Considering chronic effect, quantitative data of all parameters were performed by repeated measures of ANOVA with fixed factors “product”, “period”, “subject” and baseline. Considering acute effect, quantitative data for all parameters were performed by repeated measures of ANOVA on TAUC with fixed factors “product”, “period” and “subject”.

The statistical analyses were performed with the use of SAS 9.1.3 Service Pack 4 software (SAS institute, Cary, NC, USA). Data are considered significant when p was less than 0.05.

Whenever data were not normally distributed, as for plasma and VLDL TG, a log transformation was applied before statistical analysis.

## Results

### Water composition

SY and referent water composition are presented in Table 1: Compared to referent water, SY water is very mineralized (20 fold more: 79 versus 4 mmol/L) and is essentially richer in bicarbonates (25 fold), sodium (50 fold) and potassium (120 fold). Chlorides, sulfates and calcium content is respectively higher by 7, 10 and 2 fold in SY than referent water.

### Baseline characteristics of study participants

There were no significant differences in anthropometric data between the subjects of the two sequences of water consumption (Table 2). After the washout period the baseline values were not different for all parameters.

### The comparison between the two waters

Statistical analysis showed no difference for all the measured parameters between the two waters (Tables 4 and 5).

**Table 4:** Glycemia, blood pressure, plasma and VLDL TG and total, VLDL, HDL and LDL cholesterol levels in fasting state (g/L) at V1 and V2 in participants after consumption of referent or SY water

			**V1 (n = 12)**	**V2 (n = 12)**	**p-value**
glycemia g/L	T0	SY	0.928 ± 0.064	0.908 ± 0.059	NS
		Referent	0.968 ± 0.085	0.931 ± 0.067	NS
blood pressure (mmHg)	Systolic	SY	121.6 ± 17.6	129.8 ± 8.2	NS
		Referent	119.8 ± 13.3	121.8 ± 10.0	NS
	Diastolic	SY	73.2 ± 14.8	77.5 ± 9.0	NS
		Referent	79.7 ± 13.6	75.9 ± 9.9	NS
Plasma TG (g/L)	T0	SY	1.217 ± 0.536	0.940 ± 0.492	p < 0.01
		Referent	1.12 ± 0.59	1.01 ± 0.38	NS
plasma cholesterol (g/L)	T0	SY	2.39 ± 0.26	2.35 ± 0.20	NS
		Referent	2.22 ± 0.0.35	2.32 ± 0.25	NS
VLDL TG (g/L)	T0	SY	0.767 ± 0.347	0.529 ± 0.355	p < 0.01
		Referent	0.670 ± 0.427	0.619 ± 0.289	NS
VLDL cholesterol (g/L)	T0	SY	0.353 ± 0.103	0.298 ± 0.100	p = 0.066
		Referent	0.338 ± 0.160	0.318 ± 0.090	NS
HDL cholesterol (g/L)	T0	SY	0.414 ± 0.105	0.460 ± 0.124	NS
		Referent	0.425 ± 0.159	0.445 ± 0.128	NS
LDL cholesterol (g/L)	T0	SY	1.282 ± 0.172	1.339 ± 0.184	NS
		Referent	1.290 ± 0.201	1.277 ± 0.199	NS

Mean±SD, n=12.

**Table 5:** Total area under the curve for glugose, plasma and VLDL TG, VLDL, HDL and LDL cholesterol during postprandial state at V1 and V2 in participants after consumption of referent or SY water

		**V1 (n = 12)**	**V2 (n = 12)**	**p-value**
Glycemia (g/L.min)	SY	428.93 ± 28.69	440.37 ± 25.64	NS
	Referent	423.59 ± 25.05	432.83 ± 27.58	NS
Plasma TG (g/L.min)	SY	839.08 ± 397.06	637.43 ± 317.36	NS
	Referent	781.66 ± 362.94	679.03 ± 250.25	NS
VLDL TG (g/L.min)	SY	487.14 ± 233.33	383.39 ± 219.80	NS
	Referent	480.55 ± 252.15	419.15 ±165.28	NS
VLDL cholesterol (g/L.min)	SY	171.99 ± 57.77	163.59 ± 46.04	NS
	Referent	167.28 ± 60.39	171.47 ± 47.67	NS
HDL cholesterol (g/L.min)	SY	192.41 ± 50.31	217.38 ± 59.41	NS
	Referent	200.54 ± 66.87	213.50 ± 67.47	NS
LDL cholesterol (g/L.min)	SY	604.56 ± 70.14	634.28 ± 85.18	NS
	Referent	620.03 ± 111.59	604.67 ± 69.00	NS

(TAUC_T0-480_(g/L.min)), mean±SD, n=12.

No significant variation was reported on arterial tension (systolic and diastolic blood pressure, considering SY or referent waters consumption in basal or postprandial state (Tables 4 and 5).

Glycemia, blood pressure, TG and cholesterol concentrations in plasma and lipoprotein at baseline (T0) and in postprandial state (TAUC_T0-T8h_) are presented in Table 4 and Table 5 respectively for subjects before and after SY or referent water consumption. No significant effect was shown when subjects consumed the low-mineralized referent water.

### Effect of SY water on lipid parameters

At fasting state, when subjects ingested SY water, we observed a significant decrease in plasma TG (23%, p < 0.01) and in VLDL TG (31%, p < 0•01) and a tendency to a decrease of VLDL cholesterol (16%, p = 0.066) (Table 4).

At postprandial state, the results showed no significant difference neither for plasma and VLDL TG nor for VLDL, LDL and HDL cholesterol (Table 5).

## Discussion

Although, no difference was observed between the two waters in this pilot study, we showed that the consumption of high mineral water (SY) during eight weeks in moderated hypercholesterolemic subject induced a decrease in plasma and VLDL TG and tended to decrease VLDL cholesterol. This high mineral water did not affect lipids parameters at postprandial state.

In our study, no change in blood pressure was observed. Hypertensive effect of sodium has been noted when accompanied by chloride but not by bicarbonate [23-25]. This explains that, although mineralized water of our study provided 2 grams per day of sodium, no change in blood pressure was noted as this water provided 5 g of bicarbonate but only 0.4 g of chloride.

At the postprandial state, plasma TG, VLDL TG and chylomicron TG concentrations were not affected by water consumption (referent or SY). In contrast to our study, previous data, from the same team, reported an improvement of lipidic charge in postprandial state by water rich in bicarbonate [15,19]. The absence of effect of bicarnonated water on postprandial lipemia in our study was unexpected and could be explained by the lower energetic meal provided in our study (1089 Kcal in the two studies vs 799 Kcal in ours). Although we have used a meal rich in fat (42% VS 36%) to stimulate postprandial lipemia, this lipidic charge was not sufficient to induce a higher postprandial triglyceridemia. In fact, the elevated TG that we measured during postprandial test, whatever the water consumed, is lower than what is classically reported [15,26,27]. Studies investigating postprandial lipemia provided more than 1000 Kcal and at least 60% of calories from fat [16,27]. Thus we suppose that the increase of lipidic charge in postprandial test could induce a significant hypertriglyceridemia during meal absorption and higher probability to measure a bicarbinated water effect. Moreover a strong association has been reported between postprandial triglyceridemia and early atherosclerosis markers [6,7,28] which suppose that fasting triglyceridemia is tightly dependent on the postprandial lipemia. Indeed, as soon as 1979, Zilversmit proposed that atherosclerosis was a postprandial phenomenon [29].

In the present study SY chronic ingestion induced a decrease in plasma TG related to a diminution of VLDL TG. We also reported a tendency to decrease VLDL cholesterol. The improvement of plasma lipid and cardiovascular risk by chronic ingestion of a high bicarbonate water was also reported by other studies [13-15] with differential effect on lipoproteins. Some studies reported a decrease in LDL cholesterol [13,14] and others showed a decrease in LDL cholesterol associated with an increase in HDL cholesterol [15] were reported. In this series of experiments, effect on TG was observed only in postprandial state while no change was observed in fasting state. One probable mechanism that could explain the decrease of VLDL TG in our work is a postprandial lipemia effect, although not observed in our study. As discussed higher a strong association has been reported between postprandial triglyceridemia and fasting lipid levels [30]. In fact, intestine derived lipids during absorption modulates liver VLDL synthesis and secretion which could affect triglyceridemia measured in fasting state [31-33].

These different results showing effect on cholesterol in almost studies [13,15,19] and on triglycerides in our case, could be related to the mineral composition of used waters. SY water is characterized by, a higher mineralization compared to the others studies and among anions a higher concentration in bicarbonate and almost a lower one in chloride. SY is water richer in bicarbonated (4167 mg/L) and less rich in chloride (329 mg/L) and contains a very high molar ratio HCO3/Cl (7.3). We did not observe any effect on LDL cholesterol, potentially described as bile acid dependent [13] but we observed an effect on plasma and VLDL TG. Effect on plasma cholesterol and bile acid excretion has been initially observed in the pioneer study of Capurso *et al*., [13]. In this later study, authors have used Montecatini water witch correspond to a low bicarbonate (677 mg/L) and low chloride content (922 mg/L) and then a very low molar ratio HCO_3_/Cl (0.43). The same effect was recovered in the studies of Vaquero’s laboratory [14-16] using a water (Vichy-Catalan) more bicarbonated (2094 mg/L) but even rich in chloride (583 mg/L) and then an intermediate molar ratio HCO_3_/Cl (2.1). It is well known that chloride and bicarbonate have differential effects on vascular parameters [24,25,34]. It has been also shown that a high concentration of NaCl increased gallbladder motility [35] that could be related to bile acid excretion. Thus it could be credible to hypothesize that bicarbonated waters with a high Cl/HCO_3_ ratio could affect plasma cholesterol by the stimulation of bile acid excretion, while water with low Cl/HCO_3_ ratio affect mainly TG concentration by affecting intestinal absorption.

## Conclusion

In conclusion, although this study did not show any difference between the two waters, data comparison before and after sodium bicarbonated mineral consumption showed a specific decrease in plasma and VLDL TG at basal state. Unfortunately we measured no effect on lipid kinetics during postprandial phase probably due to our meal test that was not rich enough in lipids. Originality of the effects observed in lipid parameters by SY water used in our study could be related to its specific mineral composition (high mineralization with high bicarbonate concentration and low chloride concentration). These promising results raised a number of questions debated in the manuscript which could be confirmed by future study with a larger number of subjects presenting elevated triglyceridemia as in particular metabolic syndrome patient.

## Competing interests

The authors declare that they have no competing interests.

## Authors’ contributions

YZ contributed to the conception and the design of study and conducted the clinical trial; Biofortis provided technical assistance; KO conceived the study, participated in the design and wrote the first version of manuscript. BH and FKC participated to the manuscript writing and revision, MP: study coordinator. All authors have contributed to the revision of the submitted manuscript and agreed with this version.
